# ‘Student tutors go online’ - Investigation of cognitive and social congruence in online student tutorials - a longitudinal study

**DOI:** 10.1080/10872981.2022.2100038

**Published:** 2022-07-10

**Authors:** Teresa Loda, Nils Berner, Rebecca Erschens, Christoph Nikendei, Stephan Zipfel, Anne Herrmann-Werner

**Affiliations:** a Tübingen Institute for Medical Education, University of Tuebingen, Tuebingen, Germany; b Deanery of Students’ Affairs, University’s Faculty of Medicine, Tuebingen, Germany; c Department of Psychosomatic Medicine and Psychotherapy, University Hospital Tuebingen, Tuebingen, Germany; d Centre for Psychosocial Medicine, Department of General Internal Medicine and Psychosomatics, University Hospital Heidelberg, Heidelberg, Germany

**Keywords:** peer-assisted learning, cognitive congruence, social congruence, online teaching, student tutorials

## Abstract

The concept of peer-assisted learning (PAL) has been implemented at many medical faculties. Due to the Covid-19 pandemic, parts of the medical education experience transitioned to digital formats. However, little is known about PAL and online student tutorials. PAL is effective due to cognitive and social congruence. This study aims to investigate these concepts in an online student tutorial on taking a patient’s medical history. This longitudinal study took place in a preclinical communication course on how to take a patient’s medical history. In an online student tutorial, the students learned how to take a patient’s psychosocial medical history. Using standardised questionnaires, cognitive and social congruence were assessed. T-tests of independent samples were performed for data comparison. The participants included 128 second-year medical students and 5 student tutors. Cognitive congruence (M_student_ = 4.19 ± 0.56; M_studenttutor_ = 4.04 ± 0.57) and social congruence (M_Student_ = 4.25 ± 0.56; M_Studenttutor_ = 4.06 ± 0.57) were high for both students and student tutors in the online setting. In comparison to the face-to-face group, students in the online setting considered the student tutors to be significantly (p < .05) more socially congruent. Learning success increased during the course; however, it was not influenced by cognitive congruence. Cognitive and social congruence are high in an online setting. The students’ learning success increased during the online tutorial. Based on the higher level of social congruence, student tutors might be very motivated to be open and approachable in an online setting. Simultaneously, students might pay more attention and participate actively in the online setting. Social and cognitive congruence contribute to the effectiveness of online student tutorials and, thus, online student tutorials should be integrated into medical training.

## Introduction

Student tutorials are a significant part of medical education. The concept of peer-assisted learning (PAL) has been implemented at many medical faculties [1]. PAL represents a favored method in university teaching as medical students are taught by their peers [2]. PAL aims to reduce distance between student and course content so that students are taught by other students who previously attended the course they are taking. Peers are closer to students, understand their difficulties better and can explain topics at an appropriate level [3–5]. Thus, PAL takes place via student tutorials to complement the usual medical curriculum and is implemented in various settings and subjects, such as problem-based learning, skills labs, anatomy, history taking and communication skills [6–9].

### Cognitive and social congruence in PAL

Several studies have shown that PAL is especially effective when student tutors and students perceive themselves as cognitively and socially congruent [3,4,10,11]. Cognitive congruence is defined as the dynamic when student tutors and students have the same knowledge framework [3,4,10,11]. In comparison to lecturers, student tutors also use language that is more familiar to students [3,11–13]. Thus, student tutors better understand where students struggle and can explain topics at an appropriate level [3–5,12]. Social congruence is created by the fact that student tutors and students share the same social roles, e.g., they are both medical students [3,4,13]. Student tutors are considered socially congruent when they are interested in students’ daily life and when they behave supportively and empathically towards students [3,4,14]. Furthermore, when they are socially congruent, student tutors encourage their students to actively participate in class by giving feedback, taking risks and asking questions [10,15,16].

### Online teaching

Due to the Covid-19 pandemic, classroom lectures, seminars, and clinical placements could no longer be performed in the usual face-to-face settings and needed to transition to a digital setting. Even practical exams like OSCEs began to take place online via video conference systems [17]. Based on the results of a national cross-sectional survey on online teaching in the UK, Dost et al. (2020) suggested that medical schools could increase the efficacy of medical education by combining face-to-face and online teaching that was focused on team-based or problem-based learning [18]. In a study by Loda et al. (2020), when medical students were asked about their expectations regarding Covid-19 and medical education, they considered the digital transformation of medical education to be a change that may improve the medical curriculum by allowing students to complete the curriculum with virtual or augmented reality [19]. Student tutorials also needed to shift to a digital format. Co et al. (2021) investigated the clinical competency of basic surgical skills in an online vs. face-to-face tutorial and found no significant difference in clinical competency after attending the different course formats [20]. However, when it comes to online tutoring, the question arises whether the cognitive and social congruence underlying fruitful learning processes might be affected by the online format.

### Aim

This study aimed to investigate cognitive and social congruence between students and student tutors in online student tutorials. Furthermore, the study investigated the relationship between students’ learning success and cognitive and social congruence in the online student tutorials.

## Material and methods

### Study design

This study presents a longitudinal quantitative design focusing on cognitive and social congruence in online student tutorials and their influence on students’ learning success. Second-year medical students were invited to take part in the study. It took place at the Medical Faculty of the Eberhard-Karls University of Tübingen in the 2021 summer term (from April to July 2021).

### Ethics

The study received ethics approval from the Ethics Committee of Tübingen Medical Faculty (no. 162/2021BO2). Participation was voluntary, and students did not receive any reimbursement for participating. All participants provided their written informed consent, and all of their responses and data were kept anonymous.

### Measurements

Cognitive and social congruence were assessed by the reliable and valid questionnaire of Loda et al. (2020) [21]. Cronbach’s Alpha of cognitive congruence was 0.817 for students and 0.842 for student tutors, and Cronbach’s Alpha of social congruence was 0.913 for students and 0.927 for student tutors [21]. Cognitive congruence was assessed by 7 items and social congruence by 15 items, both rated on a 5-point Likert scale ranging from 1 (I strongly disagree) to 5 (I strongly agree). Measures of cognitive congruence were similar to questions such as ‘The tutor was able to explain issues adapted to the students’ language and knowledge’ or ‘The tutor asked students questions they were well able to understand.’ Social congruence was measured with items such as ‘The tutor proved to be interested in me as student and learner’ or ‘There was a supporting and trustful learning basis between tutor and students’. The questionnaire was separated into students’ and student tutors’ view. The medical students were also asked about possible technical difficulties like internet connection or video transmission and how actively they participate in class. To measure learning success, students were asked to complete a checklist where they named the relevant aspects of a patient’s psychosocial medical history.

### Procedure

This study was integrated into a regular communication training called ‘iTüpFerl’. In the iTüpFerl course, students practice basic skills in communication, interacting with a patient in different communication settings with an increasing degree of fidelity, including role plays and encounters with standardised patients and real patients. The study took place on the second practice unit, where medical students have to take the psychosocial medical history. This practice unit represented one session and lasted 60 min. In this practice unit, the medical students take the psychosocial medical history with a standardised patient wearing an obesity suit simulating a patient suffering from diabetes [22]. The medical students should find out possible psychosocial factors for why the patient cannot handle his diabetes. The practice unit was taught by a student tutor. The student tutors received a didactic and thematic training before the teaching. The students were divided into groups based on their allocation of the Medical Faculty. The student tutors were evenly randomised to the several groups.

At the end of the practice unit, all medical students, including the student tutor, evaluated the cognitive and social congruence.

**Figure 1.**
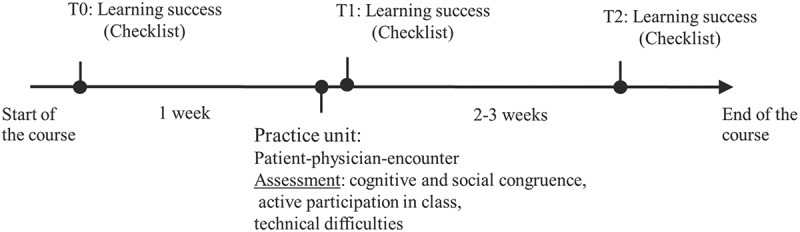
Study design and process of the study. At the start of the course, the first learning success check took place. One week later, the students participated in the online practice unit (60 min), followed by the second learning success check. After two to three weeks, the third learning success check took place.

To measure learning success, the medical students were asked to complete the checklist at three different time points: at the start of the communication course (T0), in the practice unit after the patient-physician encounter (T1) and towards the end of the communication course (T2). Please see Figure 1 for more details.

### Data analysis

The normal distribution of the data was confirmed using the Kolmogorov–Smirnov test. Descriptive data, including mean values (M), standard deviations (SD), sum scores, frequencies and percentages of relevant factors, were calculated, and any missing value was replaced with the mean value. To compare the results of cognitive and social congruence between student tutors and students, *t*-tests for independent samples were performed. Moreover, effect sizes of Cohen’s d were calculated for cognitive and social congruence. The results of cognitive and social congruence were also compared to the face-to-face group of Loda et al. (2020), which presented raw data in a face-to-face setting by using *t*-tests for independent samples [21]. The raw data of the face-to-face setting consisted of 676 medical students from several years of study (including first until last year of study) of the Medical Faculty of University of Tuebingen collected in 2018 [21].

Learning success was compared by using*t*-tests for dependent samples over the three time points. To test the influence of cognitive and social congruence on learning success, the Likert scale of the questionnaire for cognitive and social congruence was transformed from 0 to 4 to 1 to 5. Furthermore, correlations were calculated using Pearson and Spearman correlations. The Statistical Package for the Social Sciences version 26.0 (IBM, Armonk, NY, USA) was used for data analysis. The level of significance was set to p*<* .05.

## Results

### Sample

Of 165 medical students invited to participate, 128 took part in the study (Response Rate = 77.6%). The average age was 22.64 ± 2.44, with 67.7% of participants being female. Five student tutors participated in the study, having an average age of 25.60 ± 3.26 and 80% of whom were female.

### Cognitive congruence

The medical students evaluated the student tutors as being cognitively congruent, with M = 4.19 ± 0.56. The student tutors likewise considered the students to be cognitively congruent, with M = 4.04 ± 0.57. When regarding the single items of cognitive congruence, there were significant differences for *similar language* as well as *stress-free and relaxed learning atmosphere*, where the students agreed significantly higher in comparison to the student tutors. Please see Table 1 for more details.

**Table 1.** Cognitive congruence from the students’ and student tutors’ perspectives in online student tutorials. Items that differ significantly are marked with *. The data were collected in summer term 2021 at the Medical Faculty of the Eberhard-Karls University Tuebingen, N = 128 medical students participated.

	Medical students		Student tutors		t-test	Cohen’s d
Items	Mean	SD	Mean	SD	p	d
Shared knowledge base	3.52	1.00	3.71	0.64	0.256	0.209
Similar language use*	4.36	0.74	4.00	0.71	0.042	0.498
Tutor prefers informal contact	3.78	1.08	3.81	0.93	0.901	0.030
No hesitation in case of ambiguities	4.31	1.01	3.95	0.921	0.133	0.364
Stress-free and relaxed learning atmosphere*	4.49	0.80	4.05	1.07	0.031	0.525
Comprehensible and informal communication	4.46	0.79	4.14	0.79	0.092	0.409
Open and non-judgmental learning environment	4.40	0.87	4.00	0.78	0.051	0.474

### Social congruence

Overall, the medical students considered the student tutors to be socially congruent (M = 4.25 ± 0.56), and the student tutors also perceived the students as socially congruent (M = 4.06 ± 0.57). However, there were significant differences for single items of social congruence, including *being empathic, effectiveness in group discussion, being open for questions, being interested in the students* and *supportive and trustful learning base*. The student tutors agreed significantly more that they were interested in the students and their daily lives. In the other items that were significantly different, the students were more likely to agree than the student tutors (see Table 2.)

**Table 2.** Social congruence from the students’ and student tutors’ perspectives in online student tutorials. Items that differ significantly are marked with *. The data were collected in summer term 2021 at the Medical Faculty of the Eberhard-Karls University Tuebingen, N = 128 medical students participated.

	Medical students		Student tutors		t-test	Cohen’s d
Items: The tutor …	Mean	SD	Mean	SD	p	d
was interested in our problems	4.54	0.70	4.43	0.68	0.527	0.153
helped us understand the topics	4.52	0.72	4.19	0.60	0.056	0.463
could explain the topics using my language and knowledge	4.56	0.72	4.33	0.66	0.194	0.314
took time to answer our questions	4.62	0.68	4.57	0.51	0.777	0.068
supported us when facing difficulties with the subject matter	4.54	0.75	4.33	0.58	0.246	0.280
showed empathy by responding to my expectations and needs*	4.56	0.67	4.14	0.73	0.013	0.604
showed interest in me as a student and learner	4.39	0.84	4.19	0.75	0.308	0.246
was able to mediate effectively in group discussions*	4.20	0.93	3.33	0.66	< 0.001	0.980
was open and accessible to questions and problems*	4.69	0.58	4.38	0.59	0.031	0.524
gave me helpful and constructive feedback	4.59	0.73	4.48	0.60	0.521	0.155
considered the student tutor as role model	3.45	1.12	3.52	0.93	0.79	0.064
was interested in me and my everyday life*	3.14	1.21	3.86	1.24	0.016	0.588
a supportive and trusting learning environment developed between the tutor and me*	4.32	0.81	3.90	0.94	0.038	0.504
I was relaxed as the student tutor had already passed the course	3.48	1.24	3.67	1.16	0.538	0.148
Overall, the student tutorial was effective	4.15	0.84	4.10	0.70	0.775	0.069

For both medical students and student tutors, a significant high correlation was measured regarding cognitive and social congruence (students: r_pearson_ = 0.686, p < .001; student tutors: r_spearman_ = 0.771, p < .001).

### Comparison with face-to-face group

**Figure 2.**
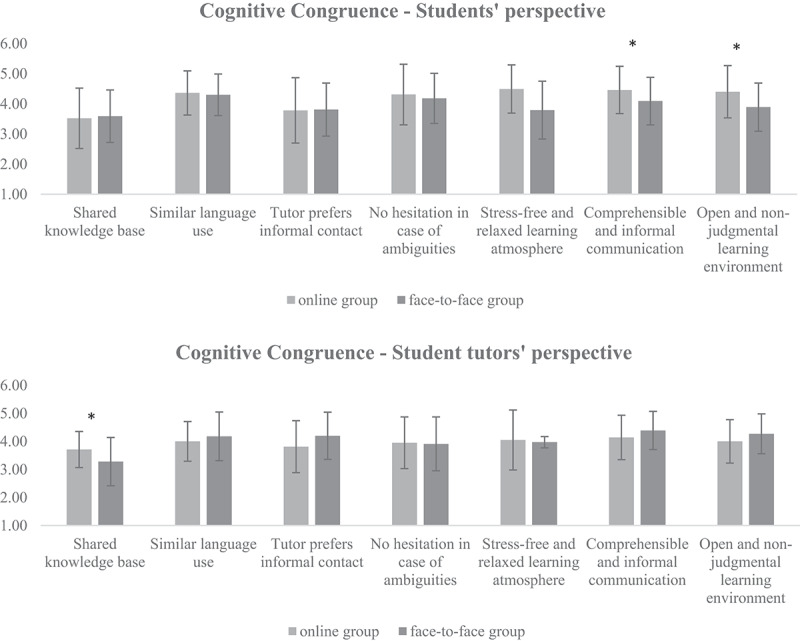
Cognitive congruence from the students’ and student tutors’ perspectives in comparison to the face-to-face group. Items that differ significantly with p < .05 are marked with *. The data were collected in summer term 2021 at the Medical Faculty of the Eberhard-Karls University Tuebingen, N = 128 medical students and five student tutors participated.

**Figure 3.**
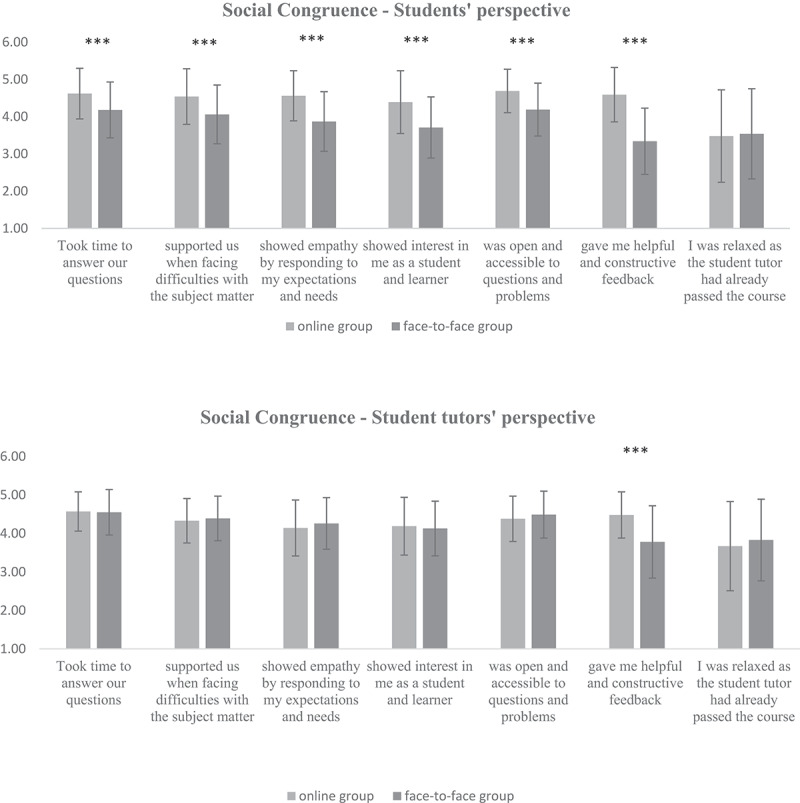
Social congruence from the students’ and student tutors’ perspectives in comparison to the face-to-face group. Items that differ significantly with p < .001 are marked with ***. The data were collected in summer term 2021 at the Medical Faculty of the Eberhard-Karls University Tuebingen, N = 128 medical students and five student tutors participated.

When comparing the above results in the online setting with a face-to-face group [21], similar scores were found for cognitive congruence for medical students (M_face-toface_ = 4.01 ± 0.54 vs. M = 4.19 ± 0.56, p > .05) as well as for student tutors (M_face-toface_ = 4.15 ± 0.53 vs. M = 4.04 ± 0.57, p > .05). The social congruence results for student tutors were also similar (M_face-toface_ = 4.23 ± 0.50 vs. M = 4.06 ± 0.57, p > .05). The medical students’ social congruence in the online setting was significantly higher than the face-to-face group (M_face-to-face_ = 3.83 ± 0.60 vs. M = 4.25 ± 0.56, p < .05). Please see Figure 2 and Figure 3 for more details.

When regarding the single items for social congruence, the medical students in the online setting scored significantly higher in comparison to the face-to-face group, indicating that social interactions such as showing empathy and interest or giving feedback were not affected by the online format. The student tutors in the online format also gave significantly higher scores on the items related to being interested and giving feedback. As for cognitive congruence, the medical students indicated significantly higher ratings for items related to a stress-free and relaxing learning atmosphere and an open learning environment. However, the student tutors in the online tutorial agreed significantly less that the contact was informal when compared to the tutors in the face-to-face group. Please see the Table 3 in the Appendix for all results.

### Learning success

The students were asked to complete the checklist for psychosocial medical history at three different time points. The results of these checklists indicate a significant increase (p < .05) in learning success. Medical students completed the checklist with fewer mistakes as time went on, reaching the highest score at the last measuring point, T2 (M_T0_ = 3.65 ± 2.61; M_T1_ = 4.55 ± 2.24; M_T2_ = 5.53 ± 2.3; T0/T1: Cohen’s d = 0.36 [low]; T0/T2: Cohen’s d = 0.74 [high]; T1/T2: Cohen’s d = 0.41 [moderate]). However, there was no significant correlation between cognitive congruence and learning success.

### Students’ perception of study environment and evaluation of the course

Among the medical students, 76.1% participated in the online tutorial at their desk and 15.4% in their office, and 83.8% had no technical difficulties. None of the student tutors had technical difficulties. Furthermore, the medical students reported that they were not disturbed by external factors like children or noise (M = 3.07 ± 2.20). However, 33.3% admitted that they were distracted by other things like email or social media. Simultaneously, the medical students agreed partly that they participated actively in the communication course just as they would in a face-to-face format (M = 3.05 ± 1.31). However, the medical students did not prefer the online format over the face-to-face setting (M = 2.05 ± 1.17).

### Discussion

This study investigated cognitive and social congruence in online student tutorials and their influence on learning success.

The results showed that students considered student tutors to be cognitively and socially congruent in online tutorials. Similarly, student tutors perceived the medical students as cognitively and socially congruent. In comparison to the face-to-face group, medical students in the online tutorial rated social congruence significantly higher but cognitive congruence about the same. Regarding learning success, students scored higher on the checklist of psychosocial medical history as the communication course went on. However, learning success was not influenced by cognitive congruence. Moreover, the medical students and student tutors had no technical difficulties during the tutorial.

The current literature or data availability regarding the subject of the study are scarce. Thus, the discussion is limited to current findings and we tried to compare the results with previous findings in a face-to-face setting.

### Cognitive congruence and social congruence in online student tutorials

Overall, medical students perceived student tutors as cognitively and socially congruent. The student tutors likewise considered the students to be cognitively and socially congruent. Due to the Covid-19 pandemic, the communication course could not take place in person, and no direct comparison to a face-to-face condition was available. Thus, we decided to compare the data from the online communication course with raw data from a face-to-face group [21]. There was no significant difference in cognitive congruence between the online setting and the face-to-face group for either students or student tutors. Similar results were found for social congruence from the student tutors’ perspective. However, the students rated social congruence higher in the online setting in comparison to the face-to-face group. This result was also found in a pilot study where cognitive and social congruence were compared in student tutorials in an online setting versus a face-to-face setting [23]. In the present study, several items of social congruence like *helping students, being interested* and *being open and approachable* were rated higher in the online setting. Student tutors are often highly motivated and enthusiastic about their job as they want to share their knowledge and teach students the relevant skills [3,24,25]. At the beginning of the Covid-19 pandemic, student tutorials were not allowed to take place due to social distancing. While other teaching concepts like lectures or clinical courses were converted into a digital format, student tutorials were left out. This study provided the student tutors an opportunity to teach again, perhaps making them highly motivated and enthusiastic about contributing to social congruence. Furthermore, Stephens et al. (2016) indicated that student tutors’ motivation might come along with cognitive congruence [26]. As cognitive congruence was high, the student tutors might have been motivated to rise to the challenge of the new online setting by remaining open, approachable and empathetic.

Furthermore, Coetze et al. (2018) found that attendance of web conference-based tutorials positively impacted students’ academic performance, with the majority of respondents agreeing that regularly attending the web conferences and connecting with instructors improved their academic performance [27]. Based on these results, the question arises as to whether students might be more likely to pay attention in an online setting. The results of the current study showed that the students were focused in the online student tutorial, as they participated actively, were not distracted and had no technical difficulties. The students might have focused on the student tutors’ behavior in the online setting, thus rating the student tutors as being more socially congruent. Future research should focus on the students’ cognitive and social processes when participating actively in online student tutorials.

### Learning success

The students’ learning performance increased as the communication course went on, implying that student tutors were effective teachers and might be as good as professional lecturers [3,25,28]. However, contrary to previous literature, learning performance was not influenced by cognitive congruence as several studies have reported [3,11,16]. This result suggests that the online setting prevented cognitive congruence from contributing to learning success.

### Strengths and limitations

To the best of our knowledge, this study presented the first investigation of cognitive and social congruence in online student tutorials. Limitations of our study are that we had a limited number of participants, with just 5 student tutors, and that they might have been particularly motivated when participating in the study. The missing randomisation of participants also presents a limitation. However, we think that it does not impact the results as we followed the allocation of the Medical Faculty. Furthermore, this study considered only how to teach one soft skills like taking medical history from a patient with non-controlled diabetes. Moreover, soft skills might be easier to teach than hard skills. Thus, future research should consider the teaching of further scenarios on how to take a patient’s medical history as well as the teaching of hard skills in an online setting.

### Conclusion

This study investigated cognitive and social congruence in an online communication course. The results showed that cognitive and social congruence were high for both the students and the students tutors. In comparison to the face-to-face groups, the students rated social congruence significantly higher in the online format, implying that student tutors were very motivated to be open and approachable with the students. Simultaneously, students might pay more attention in the online setting when participating actively, which may lead them to consider the student tutors to be more socially congruent. Future research should focus on the cognitive and social processes in online student tutorials. Regardless, the results showed that online student tutorials are effective when the student tutors are motivated which contributes to social congruence and teaching soft skills. Consequently, online student tutorials should be integrated into medical training.

## Data Availability

Data are available from the corresponding editor upon reasonable request.

## Appendix

**Table 3.** Medical students’ and student tutors’ results of cognitive and social congruence compared between the face-to-face (N = 676, collected in 2018 at Medical Faculty of University of Tuebingen) and online setting (N = 133, collected in 2021 at Medical Faculty of University of Tuebingen) based on the single items.

	Medical Students					Student Tutors				
Items	Face-to-face		Online		Statistics	Face-to-face		Online		Statistics
	Mean	SD	Mean	SD	p	Mean	SD	Mean	SD	p
I1 Same knowledge base	3.59	0.87	3.52	1.00	> .05	3.28	0.86	3.71	0.64	< .05
I2 Similar language	4.30	0.69	4.36	0.74	> .05	4.18	0.87	4.00	0.71	> .05
I3 Preferring informal contact	3.81	0.88	3.78	1.08	> .05	4.20	0.84	3.81	0.93	> .05
I4 Student wasn’t afraid to tell tutor if they didn’t understand anything	4.18	0.83	4.31	1.01	> .05	3.91	0.96	3.95	0.92	> .05
I5 Being interested in students’ needs and problems	3.98	0.82	4.54	0.70	< .001	4.37	0.61	4.43	0.68	> .05
I7 Helping students	4.18	0.72	4.52	0.72	< .001	4.29	0.67	4.19	0.60	> .05
I8 Tutor was able to explain students the topics based on their language and knowledge base	4.19	1.45	4.56	0.72	< .05	4.30	0.67	4.33	0.66	> .05
I9 Taking time for questions	4.18	0.75	4.62	0.68	< .001	4.55	0.59	4.57	0.51	> .05
I10 Supportiveness of student tutor	4.06	0.79	4.54	0.75	< .001	4.39	0.58	4.33	0.58	> .05
I11 Showing empathy	3.87	0.80	4.56	0.67	< .001	4.26	0.67	4.14	0.73	> .05
I12 Being interested in student as learner	3.71	0.82	4.39	0.84	< .001	4.13	0.71	4.19	0.75	> .05
I13 Effectiveness of the student tutor	3.51	0.86	4.20	0.93	< .001	3.79	0.72	3.33	0.66	< .01
I14 Being open and approachable	4.19	0.71	4.69	0.58	< .001	4.49	0.61	4.38	0.59	> .05
I15 Helpful and constructive feedback	3.34	0.89	4.59	0.73	< .001	3.78	0.94	4.48	0.60	< .001
I18 Seeing tutor as role model	3.13	1.03	3.45	1.18	< .01	3.55	0.96	3.52	0.93	> .05
I19 Stress-free and relaxing learning atmosphere	3.79	0.96	4.49	0.80	< .001	3.97	0.2	4.05	1.07	> .05
I20 Being interested in students	3.20	1.03	3.14	1.21	> .05	3.68	0.93	3.86	1.24	> .05
I21 Easy and informal communication	4.09	0.79	4.45	0.79	< .001	4.39	0.68	4.14	0.79	> .05
I22 Trustful learning base	3.80	0.80	4.32	0.81	< .001	4.20	0.70	3.90	0.94	> .05
I23 Creating an open and non-judgmental learning environment	3.89	0.80	4.48	0.87	< .001	4.27	0.71	4.00	0.78	> .05
I24 Successfully passed the course	3.54	1.21	3.48	1.24	> .05	3.83	1.06	3.67	1.16	> .05
I26 Effectiveness of tutorial	4.09	0.83	4.15	0.84	> .05	4.36	0.73	4.10	0.70	> .05

## References

[1] Herrmann-Werner AG, Regina E, Rebecca N, et al. Peer-assisted learning (PAL) in undergraduate medical education: an overview. Zeitschrift für Evidenz, Fortbildung und Qualität im Gesundheitswesen. 2017;121:74–9. 28545616 10.1016/j.zefq.2017.01.001

[2] Vogel B, McMillan A, Dethleffsen K, et al. Peer-assisted learning – mehr als eine methode. In: Noller J,ed. Methoden in der Hochschullehre: interdisziplinäre Perspektiven aus der Praxis. Fachmedien Wiesbaden: Wiesbaden: Springer; 2019. p. 45–62.

[3] Schmidt HG, Moust JH. What makes a tutor effective? A structural-equations modeling approach to learning in problem-based curricula. Acad Med. 1995;70(8):708–714. 7646747 10.1097/00001888-199508000-00015

[4] Lockspeiser TM, O’Sullivan P, Teherani A, et al. Understanding the experience of being taught by peers: the value of social and cognitive congruence. Avances Health Sci Educ. 2008;13(3):361–372. 10.1007/s10459-006-9049-8 17124627

[5] de Menezes S, Premnath D. Near-peer education: a novel teaching program. Int J Med Educ. 2016;7:160. 27239951 10.5116/ijme.5738.3c28 PMC4885635

[6] Blohm M, Lauter J, Branchereau S, et al. Peer-Assisted Learning”(PAL) in the skills-lab–an inventory at the medical faculties of the federal republic of Germany. GMS Zeitschrift für Medizinische Ausbildung. 2015;32(1). 10.3205/zma000952. PMC4330629 25699102

[7] Burgess A, Dornan T, Clarke AJ, et al. Peer tutoring in a medical school: perceptions of tutors and tutees. BMC Med Educ. 2016;16(1):1. 26956642 10.1186/s12909-016-0589-1 PMC4784332

[8] Hall SL, Michael B, Scott, et al. Near‐peer teaching in clinical neuroanatomy. Clin Teach. 2013;10(4):230–235. 23834568 10.1111/tct.12001

[9] Hall SS, Andrade J, Davids T, et al. Perceptions of junior doctors and undergraduate medical students as anatomy teachers: investigating distance along the near‐peer teaching spectrum. Anat Sci Educ. 2014;7(3):242–247. 24170449 10.1002/ase.1419

[10] Ten Cate O, Durning S. Dimensions and psychology of peer teaching in medical education. Med Teach. 2007;29(6):546–552. 17978967 10.1080/01421590701583816

[11] Cianciolo AT, Kidd B, Murray S. Observational analysis of near‐peer and faculty tutoring in problem‐based learning groups. Med Educ. 2016;50(7):757–767. 27295480 10.1111/medu.12969

[12] Nestel D, Kidd J. Peer tutoring in patient-centred interviewing skills: experience of a project for first-year students. Med Teach. 2003;25(4):398–403. 12893551 10.1080/0142159031000136752

[13] Cameron DA, Binnie VI, Sherriff A, et al. Peer assisted learning: teaching dental skills and enhancing graduate attributes. Br Dent J. 2015;219(6):267–272. 26404990 10.1038/sj.bdj.2015.722

[14] Yew EH, Yong JJ. Student perceptions of facilitators’ social congruence, use of expertise and cognitive congruence in problem-based learning. Instructional Sci. 2014;42(5):795–815.

[15] Chng E, Yew EH, Schmidt HG. To what extent do tutor-related behaviours influence student learning in PBL? Adv Health Sci Educ. 2015;20(1):5–21. 10.1007/s10459-014-9503-y 24740140

[16] Tayler N, Hall S, Carr NJ, et al. Near peer teaching in medical curricula: integrating student teachers in pathology tutorials. Med Educ Online. 2015;20(1):27921. 26134584 10.3402/meo.v20.27921 PMC4488334

[17] Gulati RR, McCaffrey D, Bailie J, et al. Virtually prepared! Student-led online clinical assessment. Educ Primary Care. 2021;32(4):245–246. 33843480 10.1080/14739879.2021.1908173

[18] Dost S, Hossain A, Shehab M, et al. Perceptions of medical students towards online teaching during the COVID-19 pandemic: a national cross-sectional survey of 2721 UK medical students. BMJ Open. 2020;10(11):e042378. 10.1136/bmjopen-2020-042378 PMC7646323 33154063

[19] Loda T, Löffler T, Erschens R, et al. Medical education in times of COVID-19: German students’ expectations – a cross-sectional study. PLOS ONE. 2020;15(11):e0241660. 33206678 10.1371/journal.pone.0241660 PMC7673791

[20] Co M, Chung PH-Y, Chu K-M. Online teaching of basic surgical skills to medical students during the COVID-19 pandemic: a case–control study. Surg Today. 2021;51(8):1404–1409. 33492484 10.1007/s00595-021-02229-1 PMC7829320

[21] Loda T, Erschens R, Nikendei C, et al. A novel instrument of cognitive and social congruence within peer-assisted learning in medical training: construction of a questionnaire by factor analyses. BMC Med Educ. 2020;20(1):214. 32641115 10.1186/s12909-020-02129-x PMC7346370

[22] Herrmann-Werner A, Loda T, Wiesner LM, et al. Is an obesity simulation suit in an undergraduate medical communication class a valuable teaching tool? A cross-sectional proof of concept study. BMJ Open. 2019;9(8):e029738. 10.1136/bmjopen-2019-029738 PMC6688692 31383708

[23] Loda T, Herrmann-Werner. Anne *peer-assisted learning live on air – social and cognitive congruence of student and student tutors in face-to-face vs. online student tutorial* in *Innovations of Medical Education Conference* 2020: Los Angeles 10.1080/10872981.2020.1801306 PMC7482745 32744892

[24] Bugaj TJ, Blohm M, Schmid C, et al. Peer-assisted learning (PAL): skills lab tutors’ experiences and motivation. BMC Med Educ. 2019;19(1):353. 31521146 10.1186/s12909-019-1760-2 PMC6744669

[25] Loda T, Erschens R, Nikendei C, et al. Qualitative analysis of cognitive and social congruence in peer-assisted learning - The perspectives of medical students, student tutors and lecturers. Med Educ Online. 2020;25(1):1801306. 32744892 10.1080/10872981.2020.1801306 PMC7482745

[26] Stephens JR, Hall S, Andrade MG, et al. Investigating the effect of distance between the teacher and learner on the student perception of a neuroanatomical near-peer teaching programme. Surg Radiologic Anatomy. 2016;38(1):1–7. 10.1007/s00276-016-1700-3 PMC5104784 27225186

[27] Coetzee SA, Schmulian A, Coetzee R. Web conferencing-based tutorials: student perceptions thereof and the effect on academic performance in accounting education. Accounting Education. 2018;27(5):531–546

[28] Tolsgaard MG, Gustafsson A, Rasmussen MB, et al. Student teachers can be as good as associate professors in teaching clinical skills. Med Teach. 2007;29(6):553–557. 17978968 10.1080/01421590701682550

